# Clinical Characteristics, Transmissibility, Pathogenicity, Susceptible Populations, and Re-infectivity of Prominent COVID-19 Variants

**DOI:** 10.14336/AD.2021.1210

**Published:** 2022-04-01

**Authors:** Zhen Yang, Shuo Zhang, Yu-Ping Tang, Sai Zhang, Ding-Qiao Xu, Shi-Jun Yue, Qi-Ling Liu

**Affiliations:** ^1^Key Laboratory of Shaanxi Administration of Traditional Chinese Medicine for TCM Compatibility, and State Key Laboratory of Research & Development of Characteristic Qin Medicine Resources (Cultivation), and Shaanxi Key Laboratory of Chinese Medicine Fundamentals and New Drugs Research, and Shaanxi Collaborative Innovation Center of Chinese Medicinal Resources Industrialization, Shaanxi University of Chinese Medicine, Xi’an, Shaanxi, China.; ^2^School of Public Health, Shaanxi University of Chinese Medicine, Xi’an, Shaanxi, China.; ^3^School of Clinical Medicine (Guang’anmen Hospital), Beijing University of Chinese Medicine, Beijing, China.

**Keywords:** COVID-19, SARS-CoV-2 variants, mutation, VOC, VOI, VUM

## Abstract

In addition to the rapid, global spread of SARS-CoV-2, new and comparatively more contagious variants are of considerable concern. These emerging mutations have become a threat to the global public health, creating COVID-19 surges in different countries. However, information on these emerging variants is limited and scattered. In this review, we discuss new variants that have emerged worldwide and identify several variants of concern, such as B.1.1.7, B.1.351, P.1, B.1.617.2 and B.1.1.529, and their basic characteristics. Other significant variants such as C.37, B.1.621, B.1.525, B.1.526, AZ.5, C.1.2, and B.1.617.1 are also discussed. This review highlights the clinical characteristics of these variants, including transmissibility, pathogenicity, susceptible population, and re-infectivity. It provides the latest information on the recent variants of SARS-CoV-2. The summary of this information will help researchers formulate reasonable strategies to curb the ongoing COVID-19 pandemic.

## Introduction

The coronavirus disease 2019 emerged at the end of 2019 and spread rapidly worldwide, as of 21 November 2021, there have been 256,480,022 confirmed cases and 5,145,002 deaths globally (www.who.int/publications/m/item/weekly-epidemiological-update-on-covid-19---23-november-2021). In the past year, scientists have made extensive efforts and conducted comprehensive research on the new coronavirus, to better understand its characteristics and find its vulnerabilities to control the pandemic.

**Figure 1.**
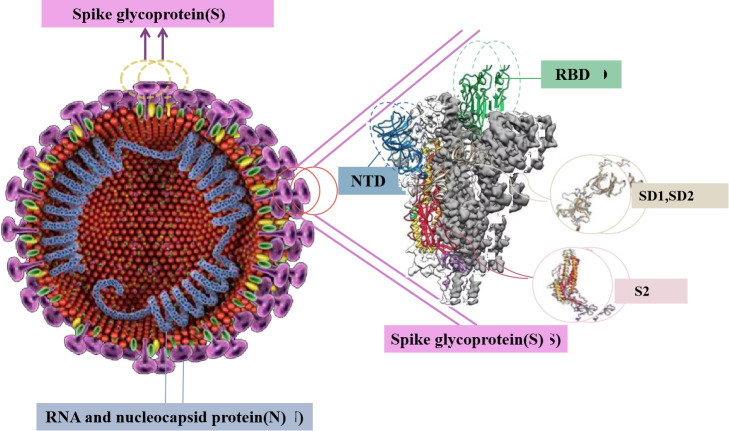
Structure of S protein in novel Coronavirus.

Coronavirus is the largest known group of viruses with forward single-stranded RNA genome [1], and it is also a member of the Coronavirus subfamily in the Coronavirus family and the Virus Order (International Committee for Classification of Viruses). According to their genome structure, this subfamily includes four species: namely α-coronavirus (α-CoV), β-coronavirus (β-CoV), γ-coronavirus (γ-CoV) and δ-coronavirus (δ-CoV) [2]. Among them, α-CoV and β-CoV typically cause human respiratory diseases and animal gastroenteritis [3]. The pathogenic viruses severe acute respiratory syndrome virus (SARS), Middle East respiratory syndrome virus (MERS), and novel coronavirus, belong to β-CoV. Among them, severe acute respiratory syndrome coronavirus 2 (SARS-CoV-2) caused the pandemic of a new coronary pneumonia worldwide. Coronavirus-invading host cells depend on the spike protein (S protein) (Fig. 1), which induces the fusion of the virus and the cell membrane by recognising the host cell receptor, such that the virus attaches to the surface of the target cell [4]. The S protein is a large type 1 transmembrane glycoprotein that is cleaved by proteolysis to form S1 and S2 [5]. S1 is responsible for target cell engagement, whereas S2 completes membrane fusion, allowing viral RNA to enter the host cell cytoplasm, where viral replication begins. S1 contains a nitrogen terminal domain (NTD) and a receptor-binding domain (RBD) that interacts with the cell receptor angiotensin-converting enzyme 2 (ACE2), which causes the new coronavirus to induce respiratory infections [6].

**Figure 2.**
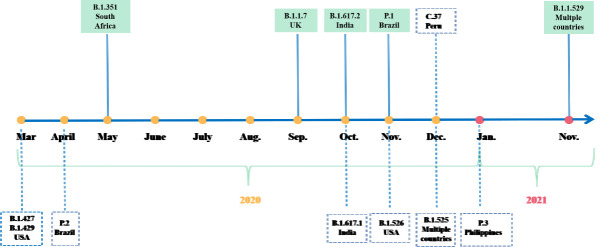
A timeline depicting the origin time of some significant variants of SARS-CoV-2. The variants of concern are marked into the green box.

**Figure 3.**
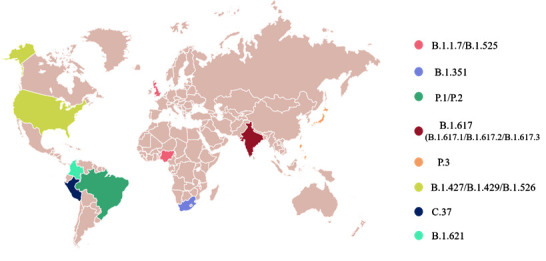
Origin of several significant variants of SARS-CoV-2.

Following the epidemic outbreak in China at the end of 2019, new mutations of SARS-CoV-2 appeared over time (Fig. 2) and geographic location. The continuous evolution of the new coronavirus has caused persistent spread of infectious mutations. The large-scale epidemic caused by this virus is difficult to control rapidly, which leads to massive virus replication and increases the chance of adaptive mutations. Among them, the mutation of a crucial protein (S protein) rendered selective advantages to the virus, which primarily manifested in improving the transmission efficiency and evading neutralising antibodies [7]. The D614G mutation in the S protein of the new coronavirus is universal [8]. This mutation increases the viral infectivity of susceptible cells by 8-10 times [9]. Subsequently, emerging lineages with spike protein mutations were first discovered in countries and regions such as the United Kingdom, South Africa, Brazil, and India (Fig. 3).

New coronavirus variants have continued to proliferate, causing close concerns about the adaptability, transmission, and disease changes of the virus. There are three naming systems for tracking and scientifically reporting the genetic evolution of new coronaviruses: GISAID, Nextstrain, and Pango. The World Health Organization (WHO) has assigned labels to the coronavirus variants according to the Greek alphabet; as of November 26, 2021, the WHO has classified the new coronavirus variants as two ‘variants of interest’ (VOIs), five ‘variants of concern’ (VOCs) (Table 1) and seven ‘Variants Under Monitoring’ (VUM). (www.who.int/en/activities/tracking-SARS-CoV-2-variants/). Five of the noteworthy variants were named Alpha (B.1.1.7), Beta (B.1.351), Gamma (P.1), Delta (B.1.617.2) and Omicron (B.1.1.529) (Table 2). The locations where these variants were first discovered were Britain, South Africa, Brazil and India, Omicron's earliest documented samples are located in multiple countries (www.who.int/publications/m/item/weekly-epidemiological-update-on-covid-19---25-may-2021). The variants have caused numerous effects on clinical, diagnostic, treatment, and public health strategies. Among patients infected with variant strains, approximately 80% had milder symptoms, which are ignored during early clinical screening. The remaining 20% of the patients developed severe ‘cytokine storm’ symptoms, which severely burdened intensive care units and increased the number of hospital beds occupied [10]. How these strains undergo a series of mutations without clear intermediate variants remains unclear; some studies have speculated that they may have evolved in immunosuppressed patients with chronic infections, and that immune plasma or monoclonal antibody (mAbs) therapy may prompt selection variants which show mutations that evade the antibody response [11].

**Table 1:** Basic characteristics of several significant SARS-CoV-2 Variants, as of Nov 1, 2021

WHO label	GISAID clade	Pango lineage	Nextstrain clade	Earliest documented samples (Time and Place)	Date of designation in WHO	Mutations
Variants of Concern (VOCs)						
Alpha	GRY (formerly GR/501Y.V1)	B.1.1.7	20I/501Y.V1	Sep-2020, United Kingdom	18-Dec-2020	69/70del, 144del, N501Y, A570D, D614G, P681H, T716I, S982A, D1118H
Beta	GH/501Y.V2	B.1.351	20H/501Y.V2	May-2020, South Africa	18-Dec-2020	D80A, D215G, 241/243del, K417N, E484K, N501Y, D614G, A701V
Gamma	GR/501Y.V3	P.1	20J/501Y.V3	Nov-2020, Brazil	11-Jan-2021	L18F, T20N, P26S, D138Y, R190S, K417T, E484K, N501Y, D614G H655Y, T1027I, V1176F
Delta	G/452R.V3	B.1.617.2	21A/S:478K	Oct-2020, India	VOI: 4-Apr-2021 VOC: 11-May-2021	T19R, G142D, E156G, F157del, R158del, L452R, T478K, D614G, P681R, D950N
Omicron	GR/484A	B.1.1.529	21K	Nov-2021 Multiple countries	VUM: 24-Nov-2021 VOC: 26-Nov-2021	-
Variants of Interest (VOIs)						
Lambda	GR/452Q.V1	C.37	21G	Dec-2020, Peru	14-Jun-2021	D614G, T859N, L452Q, F490S, T76I, G75V, R246N, 247/253del
Mu	GH	B.1.621	21H	Jan-2021, Columbia	30-Aug-2021	D614G, P681H, R346K, N501Y, T95I, E484K, D950N, Y145N, Y144S

In this study, we discuss the global variants and the properties of significant VOCs, VOIs and VUMs, and illustrate some significant variants such as D614G, N501Y, E484K, L452R, among others. Aggregating this information, we hope to help researchers formulate reasonable strategies to effectively curb the pandemic.

**Table 2:** Summary of phenotypic impacts of Variants of Concern (VOCs), as of November 10, 2021.

	Transmissibility	Severity	Impacts on different groups	Impacts on diagnostics	Risk of reinfection	Impacts on vaccines (Symptomatic disease)
Variants of Concern (VOCs)						
B.1.1.7	Increased (36%-75%)	Possible increased risk of Hospitalization, severity and mortality	All age groups	S gene target failure (SGTF), no impact on overall result from multiple target RT-PCR, No impact on Ag RDTs observed	increased secondary attack rate (10% to 13%)	VE↓, <10%: Moderna-mRNA-1273, Moderna-mRNA-1273/Pfizer BioNTech-Comirnaty, Pfizer BioNTech-Comirnaty; VE↓, <10%-20%: AstraZeneca-Vaxzevria; VE↓, <20%: Novavax-Covavax.
B.1.351	Increased [1.50 (95% CI: 1.20-2.13) times than previous strain,	Preliminary evidence suggested an association with high short-term mortality, more pathogenic than B.1.1.7	All age groups	No impact on RT-PCR or Ag RDTs observed	Reduction in neutralizing activity repor-ted; T cell respon-se elicited by D614G virus remains effective	VE↓, <10%: Janssen-Ad26. COV 2.5; VE↓, ≥30%: AstraZeneca-Vaxzevria, Novavax-Covavax.
P.1	Increased [2.60 (95% CI: 2.40-2.80) times than wild-type mutant	Related to higher viral load, Increased risk of severe infection or higher mortality (not confirmed)	Female groups and groups between 20 and 59 years old (In Amazonia)	None reported to date	Moderate reduction in neutralizing activity reported, reinfections reported	VE↓, <10%-20%: Sinovac- CoronaVac.
B.1.617.2	Increased, more transmissible than other variants	More likely to cause tissue damage and more pathogenic, Increased risk of hospitalization	All age groups	None reported to date	Reduction in neutralizing activity reported	VE↓, <10%-20%: Pfizer BioNTech-Comirnaty, VE↓, <20%: Bharat-Covaxin; VE↓, <20%-30% AstraZeneca-Vaxzevria.

*Note:* VE, Vaccine effectiveness, Vaccine effectiveness (VE) is the percentage reduction in the risk or odds of disease or infection among vaccinated persons.

## Characteristics of VOCs

### B.1.1.7 (Alpha)

On 14 December 2020, the United Kingdom reported a novel coronavirus variant (VOC) that had caused concern, lineage B.1.1.7, which is also known as VOC 202012/01 or 20I/501Y.V1 [12]. This variant first appeared in the south-eastern part of the UK in September 2020, and it soon became the main novel coronavirus variant circulating in the UK, subsequently spreading to more than 50 countries (https://virological.org/t/preliminary-genomic-characterisation-of-an-emergent-sars-cov-2-lineage-in-the-uk-defined-by-a-novel-set-of-spike-mutations/563). The vital mutations in the RBD region of this strain are E484K, S494P and N501Y, and others; other major mutations in the *S* protein are D614G, A570D, S982A, P681H, D1118H, T716I, K1191N, among others. In addition, this variant has three deletions in the *S* protein, namely, 69del, 70del, and 144del; at least three mutations may affect viral function. The mutation N501Y is a key contact residue in the RBD; it enhances the binding affinity of the virus to human ACE2. The mutation P681H is next to the furin cleavage site in the spinous process, which is a pivotal region of infection and transmission. Deletion of the spike protein DH69/DV70 in multiple independent lineages of the new coronavirus is associated with the immune escape of immunodeficient patients and enhanced the infectivity of the virus *in vitro* [13]. The DH69/DV70 deletion can be characterised by the failure to detect the *S* gene in tests, which is referred to as ‘S gene target failure’ (SGTF) [14].

Compared to other SARS-CoV-2 lineages, B.1.1.7 has higher transmissibility [15], which may be related to the following two aspects: it carries an unusually large number of specific mutations—most of which are non-synonymous and are concentrated in the *S* gene; the mutation spectrum in the *S* gene is related to certain specific functions such as immune response evasion (69/70 deletion) or enhanced affinity for the ACE2 receptor (N501Y) [16]. Kissler (2021) evaluated the longitudinal polymerase chain reaction test in 65 COVID-19 patients who underwent routine surveillance and testing, and seven of them were infected with B.1.1.7. Whole-genome sequencing demonstrated that, compared to the non-B.1.1.7 variants, B.1.1.7 may cause infection for a longer period of time at a similar peak virus concentration of the virus; this extended duration may lead to an increase in the transmission rate of B.1.1.7 [17]. Furthermore, the new strain was 56% (95% CI 50-74) more infectious than the original strain [18]; the effective reproduction number R(t) of the new strain increased by 1.4-1.8 times [19]. Calistri (2021) investigated reverse transcription polymerase chain results of nasopharyngeal swabs, tested from December 2020 to February 2021, to verify the viral load and persistence between patients infected with the B.1.1.7 lineage and other patients. Compared to people infected with other lineages (14 days), those infected with lineage B.1.1.7 (16 days) had a remarkably longer duration of the new coronavirus’ ribonucleic acid in nasopharyngeal swabs [20]. Brookman (2021) successively observed 20 children and adolescents (aged 18 years or younger and positive for SARS-CoV-2) admitted to King’s College Hospital from 1 March to 31 May 2020, and 60 new coronavirus-positive children and young people from 1 November 2020 to 19 January 2021; there were no significant differences in age, sex, and ethnicity among these patients. The results showed that children and adolescents did not have a greater number of serious diseases in the second wave of the epidemic caused by B.1.1.7, indicating that this variant infection would not cause a significantly different clinical course from the original strain [21]. Davies et al (2021). analysed the data set and linked 2,245,263 positive patients with new coronaviruses, and 17,452 new coronary pneumonia-related deaths in the United Kingdom from 1 November 2020 to 14 February 2021. Based on 4,945 deaths with known SGTF status, the risk of death associated with SGTF was 55% higher than that in cases without SGTF (95% confidence interval, 39-72%), which equated to an increase in the absolute risk of death within 28 days after a positive test for a 55-69-year-old male in the community from 0.6% to 0.9% (95% confidence interval, 0.8-1.0%). Consequently, compared to the previously existing variants of the new coronavirus, B.1.1.7 is not only easier to spread, but it may also cause more severe disease symptoms [22].

### B.1.351 (Beta)

The B.1.351 variant appeared in the Eastern Cape Province of South Africa at the end of 2020 [23] and gradually became widespread locally. The variant was isolated from an oropharyngeal swab of a patient in KwaZulu-Natal Province, South Africa, in November 2020 [24]. One hypothesis for the emergence of this lineage is that it may have evolved within the host of one or more individuals with extended viral replication [25]. The first epidemic peak of the South African mutant was from June to September; it was primarily driven by three lineages: B.1.1.54, B.1.1.56, and C.1. Tegally (2020) et al. found that the three most common variants of the 501Y.V2 lineage are 501Y.V2-1, 501Y.V2-2, and 501Y.V2-3. At the beginning of the epidemic’s second peak, 501Y.V2-1 proliferated; it could be identified by five amino acid mutations in the *S* protein (except D614G), including D80A, D215G, E484K, N501Y, and A701V. Subsequently, two more mutations appeared in the *S* protein, L18F and K417N, leading to the emergence of the 501Y.V2-2 variant; the third variant, 501Y.V2-3 was based on the deletion of *S* protein residues, Del242-244 from 501Y.V2-2 [23]. Therefore, the B.1.351 variant had 21 mutations. The important mutations in the *S* protein are D80A, D215G, 241del, 242del, 243del, D614G, and A701V. Additionally, the mutations in the RBD region are K417N, E484K, and N501Y. For context, E484K and N501Y are present in the receptor-binding motif (RBM), and the N501Y mutation has also been found in the British lineage (B.1.1.7). N501 forms a partial binding loop in the contact region of human ACE2 (hACE2) and forms with Y41 in the hACE2 hydrogen bond; it can also stabilise K353, a viral binding hotspot residue on hACE2 [26]. Concurrently, it is one of the key areas where the new coronavirus is different from SARS-CoV, which contributes to enhancing the binding affinity of the new coronavirus to hACE2. The E484K mutation is not common, and its occurrence rate is less than 0.02% in sequences outside of South Africa. K417 is a unique hACE-2 interacting residue that forms a salt bridge interaction with D30 of hACE2 in the central contact region [27], which is the most significant difference between the new coronavirus and SARS-CoV in the RBD-hACE2 complex, this helps enhance the binding affinity of the new coronavirus to hACE231-33. Moreover, in-depth mutation scanning revealed that the K417N mutation had a minimal effect on the binding affinity of hACE2 [28].

Louis (2021) conducted a retrospective single-centre study to report the preliminary observational data of 501Y.V2 patients with severe new coronary pneumonia, who were hospitalised consecutively in the intensive care unit of Moselle from 3 February to 16 March 2021. They were laboratory-confirmed to have COVID-19 and were screened for mutations. Multivariate logistic regression was used to explore the relationship between the variant strains B.1.1.7 or 501Y.V2 (V2) SARS-CoV-2 and 60-day mortality rates. V2 was highly correlated with the 60-day mortality rate (odds ratio, 5.67; 95% confidence interval, 1.04-30.81). Preliminary data indicated that the V2 variant was associated with higher short-term mortality and may be more pathogenic than the V1 strain [29]. Moreover, Pearson (2021) estimated that the B.1.351 variant strain may be more transmissible than the early circulating strains of the new coronavirus. The B.1.351 variant accounted for approximately 40% of the new SARS-CoV-2 variant infections, while the B.1.1.7 variant accounted for only 20% (https://cmmid.github.io/topics/covid19/sa-novel-variant.html). Charpentier (2021) analysed the results of 643 SARS-CoV-2 patients between 20 December 2020 and 26 February 2021. It identified that 332 historical SARS-CoV-2 patients characterised by signal detection for the three target genes and absence of the E484K mutation. 249 501Y.V1 is characterised by the lack of detection of the *S* gene and E484K mutation, and 62 501Y.V2 with signal detection for the three target genes and a signal for E484K mutation. The results showed that there were significant differences in the Ct values of ORF1ab and N target genes among the three SARS-CoV-2 variants. In fact, the new 501Y.V1 and 501Y.V2 variants have a higher nasopharyngeal viral load at the time of diagnosis than the historical lineage. Viral load of the 501Y.V2 variant appeared to be equal to or slightly less than that of the 501Y.V1 variant [30].

Furthermore, a small sample report from South Africa showed that the efficacy of the Oxford-AstraZeneca vaccine against B.1.351 infection was significantly reduced (the efficacy against mild-to-moderate diseases was 10.6%). Another randomised, double-blind, placebo-controlled, multi-site trial evaluated the safety and effectiveness of two standard doses of ChAdOx1-nCoV19: two doses of ChAdOx1-nCoV vaccine have no effect on mild-to-moderate neo-coronary pneumonia in non-hospitalised patients [31].

### P.1 (Gamma)

P.1, also known as N501Y.V3 and was first detected among four Japanese travellers returning from Amazon State, Brazil on 2 January 2021 and was quickly identified as an emerging lineage in Manaus [32]. P.1 evolved from the B.1.1.28 lineage [33]. Most cases in the Amazon region were caused by the spread of several local virus branches, which constitute 77% of the 250 Amazon genome samples of the new coronavirus between March 2020 and January 2021, rather than multiple times enter [34].

To gain insight into the antibody resistance mechanism, Wang (2021) determined the structure of the 2-proline stabilised P1 spike protein with a resolution of 3.8 A using single particle cryo-electron microscopy (cryo-EM). Overall, the structure of the P.1 spike was extremely similar to that of D614G variant [35]. In addition to D614G, P.1 also contains 10 spike mutations, including K417T, E484K, and N501Y in the RBD; L18F, T20N, P26S, D138Y and R190S in the N-terminal domain (NTD); and H655Y near the furin cleavage site. This new variant may threaten the efficacy of current monoclonal antibody (mAb) therapies and vaccines. P.1 and P.1.351 contain the same three-residue mutation in RBD, but B.1.351 K417N in P.1 is different from chromosome K417T in P.1 [35]. Three key mutations in the RBD, N501Y, K417T, and E484K, are common in B.1.1.7 and B.1.351, and they are associated with increased transmission, immune escape, and pathogenicity. N501Y and K417T interact with hACE2, E484K is located in the loop region outside the direct hACE2 interface [36]. Faria (2021) showed that three mutations in the RBD may enhance the participation of hACE2, providing a hypothesis for the increased heritability of the P.1 lineage. In addition, E484K was associated with reduced antibody neutralisation. RBD epitopes account for approximately 90% of the serum neutralising activity of patients infected with the new coronavirus virus (54); therefore, the tighter binding of P.1 virus to hACE2 may further reduce the efficacy of neutralising antibodies [37]. Prete (2021) showed that re-infections caused by P.1-induced re-infections were more common and frequent than traditional epidemiological, molecular, and genomic surveillance clinical cases [38].

The P.1 lineage is associated with a higher viral load. The estimated RE trajectory of the SARS-CoV-2 Amazon lineage supported that VOC P.1 may be more transmissible than the early epidemic virus lineage circulating in the Amazon. To test whether this estimated difference in RE would result in an evident virologic phenotype, Naveca (2021) used real-time RT-PCR cycle threshold (ct) scores as P.1 positive and P.1 negative collected at similar times from the onset of the symptoms representative of the upper respiratory tract (URT) viral load of the sample. The results showed that in URT samples from P.1 infections, the level of novel coronavirus RNA (estimated based on the median Ct) was higher than that detected in non-P.1 infections, especially in adults (18-59 years old): It was approximately 10 times higher, indicating that adult individuals infected with P.1 were more infectious than those infected with non-P.1 viruses [33]. Coutinho et al (2021). used a model to analyse the frequency of P.1 in COVID-19 hospitalisations and Manaus city residents’ sequences in Brazil’s national health surveillance data to estimate the transmission rate and the relative re-infectivity of the P.1 variant. The results showed that the estimated transmission rate of P.1 was 2.6 times higher than that of wild-type mutants (95% confidence interval: 2.4-2.8), and the estimated relative re-infectivity of the new mutant was estimated to be 0.032 [39]. Siqueira (2021) wrote a case report detailing documenting a COVID-19 family cluster related to the SARS-CoV-2 P.1 lineage. It is noteworthy that three of the five reported cases developed severe COVID-19, requiring long-term ICU treatment, and one patient died. According to the shocking increase in the number of COVID-19 deaths recently reported in Brazil, some individuals believed that the P.1 lineage may mean an increased risk of severe infection or higher mortality, but this hypothesis urgently needs further research [40].

Freitas (2021) et al. analysed the data of SIVEP-Gripe (Sistema de Informação de Vigil-ncia Epidemiológica da Gripe) when popular new variants were dominant in different periods: the first wave’s peak occurred during April to May 2020 and the second wave’s peak during January 2021. They calculated the mortality, total case fatality rate, and case fatality rate of inpatients. The results showed that after the emergence of mutant P.1 in Amazon, the proportion of deaths from COVID-19 increased in the female population and the gender population aged 20-59 years. In addition, the mortality, case fatality rate, and hospital fatality rate of different age groups and genders relatively increased [41]. This evidence suggests that P.1 had different effects on males and females in different age groups compared to previous strains, indicating changes in the pathogenicity and virulence spectrum. Naveca (2021) described three women living in Manaus, Amazonas, Brazil, respectively, who were re-infected with the new coronavirus variant P.1 in the second wave of the pandemic. Three female patients, aged 29, 40, and 50 years, were positive after two RT-PCR tests, at least 92 days apart. After genome sequencing and phylogenetic analysis, the three re-infected cases were infected with different virus lineages when they were first infected 3-9 months hitherto (cases 1 and 3: B.1.195 lineage; case 2: B.1.1.33 lineage). The viral load of the re-infected samples from Cases 1 and 2 (average Ct of 20.5 and 19.7, respectively) was higher than that of the first infection sample (average Ct of 27.5 and 34.0, respectively). In contrast, Case 3 showed roughly equivalent Ct values in the initial infection (19.9) and re-infection (21.0) samples. The symptoms of re-infection in the three patients were not as severe as the initial infection, indicating that the immune response induced by the early SARS-CoV-2 variant was sufficient to effectively prevent severe cases of COVID-19 caused by the variant P.1 [42]. The Ct value of all three patients with re-infection was low (<22), indicating that the VOC P.1 virus could effectively replicate in the nasopharynx of convalescent patients, and both symptomatic and asymptomatic reinfected individuals may be infectious [43]. A longitudinal study of health care workers showed that anti-new coronavirus IgG antibodies after infection were associated with protection against reinfection in most people for at least 6 months after infection [44]. Selhorst (2020) described a case of re-infection by a symptomatic health care worker, despite an effective humoral immune response, following a symptomatic primary (initial) infection. The patient developed short-term protective immunity after the first infection, but the anti-new coronavirus antibodies were largely absent during the second infection [45]. In a retrospective study, Silva described a 39-year-old male patient with a history of other comorbidities who was infected with the mutant strains P.1 and P.2 within 3 months. Symptoms after the first infection have not yet been reported. After re-infection, the patient experienced symptoms such as dyspnoea, fatigue, and respiratory distress. Later, due to various complications, the lung capacity was severely reduced, resulting in the patient’s death [46]. This evidence showed that although some case reports indicated that re-infection with the new coronavirus appeared as asymptomatic or mild illness, some cases develop into severe illnesses or even cause death during the second attack.

### B.1.671.2 (Delta)

The B.1.617 variant of the new coronavirus emerged in western India in October 2020, gradually spreading in various parts of India and further to the rest of the world [47]. This pedigree is derived from the B.1 pedigree (D614G) and includes three main subtypes: B.1.617.1, B.1.617.2, and B.1.617.3 (www.cdc.gov/coronavirus/2019-ncov/cases-updates/variant-surveillance/variant-info.html (2021)). The B.1.617.2 (Delta) variant is considered one of the most contagious variants. As of June 2021, it has the highest number of reported cases [48], and due to its rapid spread and potential immune evasion, it was listed by the WHO as a VOC on 11 May 2021 (https://www.who.int/publications/m/item/weekly-epidemiological-update-on-covid-19---11-may-2021). Studies have shown that compared to previous variants, the Delta variant is not only able to evade immunity from previous infections, but it is also less sensitive to neutralising antibodies from recovered patients [49]. Using *in vitro* experiments, Mlcochova (2021) proved that compared to wild-type Wuhan-1 carrying D614G, the sensitivity of B.1.617.2 to recover neutralising antibodies in the serum of individuals was approximately six times lower, and the sensitivity to vaccine-induced antibodies was approximately eight times lower [50].

The B.1.617.2 variant was isolated from a nasopharyngeal swab of a patient with a confirmed Indian life history. Compared to the D614G variant, the spike protein of the variant contains nine mutations, including five NTD mutations (T19R, G142D, δ156, δ157, R158G), two mutations in RBD (L452R, T478K), one mutation near the Flynn cleavage site (P681R), and one mutation in the S2 region (D950N) [51]. Mutations in RBD can change the ability of the viral spike protein to bind to and enter the host cell. Baral (2021) studied the effect of Delta mutation on the structure of the receptor-binding interface of RBD, as well as the RBD-ACE2 interaction and the RBD-neutralising antibody interaction. By examining the SARS-CoV-2 Ab-RBD complex available in the protein database (PDB), the differences in the RBD-Ab interaction caused by Delta variant mutations were compared, and the results showed that the Delta variant was stable but slightly recombined. The receptor-binding interfaces can lead to weakened interactions with certain neutralising antibodies, leading to immune escape [48].

**Figure 4.**
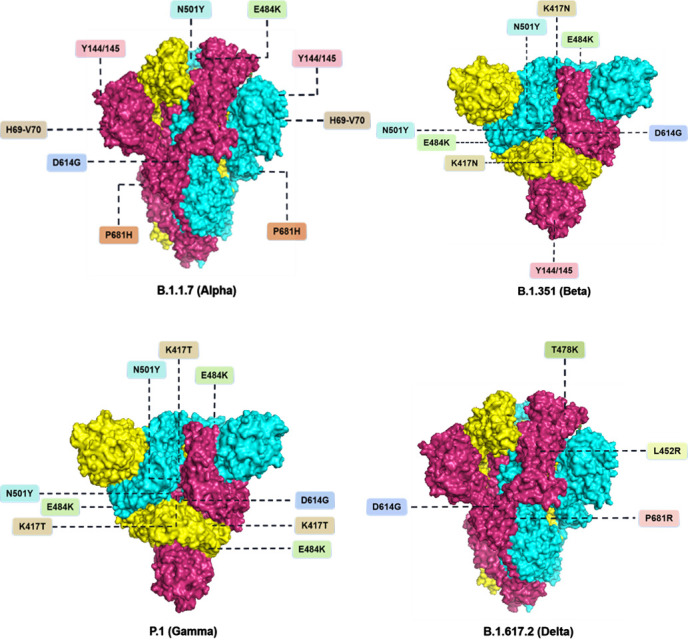
Important mutations located at the S protein in the VOCs lineage.

Syncytial formation driven by the new coronavirus *S* protein is considered one of the pathogeneses of COVID-19. Prerna (2021) studied the B.1.617.2 S protein’s ability to drive cell fusion in the human lung cancer cell line A549. The line was engineered to express high levels of ACE2, and the results showed that the targeted expression of WT-S led to syncytia formation, whereas cells transfected with the empty expression plasmid remained normal. The quantification of cell fusion showed that the fusion efficiency of B.1.617.2 *S* protein was approximately 2.5 times higher than that of WT-S. In addition, B.1.617.2 was more likely to cause tissue damage than the previous variant, which was highly pathogenic. The experiment by Arora proved that B.1.617.2 had the effects of immune escape, colon and lung cell entry enhancement, and syncytium formation, and that the B.1.617.2 *S* protein could cause more cell fusion than WT-S. This may indicate that compared to the previous variant, the B.1.617.2 protein could cause greater tissue damage and thus had a higher pathogenicity [52].

### B.1.1.529 (Omicron)

The B.1.1.529 variant was first reported to WHO from South Africa on 24 November 2021. In recent weeks, infections have increased steeply, coinciding with the detection of B.1.1.529 variant. The first known confirmed B.1.1.529 infection was from a specimen collected on 9 November 2021.

Preliminary evidence suggests an increased risk of re-infection with this variant, compared to other VOCs. Based on the evidence presented indicative of a detrimental change in COVID-19 epidemiology, the Technical Advisory Group on SARS-CoV-2 Virus Evolution (TAG-VE) (an independent group of experts that periodically monitors and evaluates the evolution of SARS-CoV-2, and assesses whether specific mutations and combinations of mutations alter the behaviour of the virus) has advised WHO that this variant should be designated as a VOC. The WHO agreed, naming the variant and the WHO has designated B.1.1.529 as a VOC, named Omicron (www.who.int/news/item/26-11-2021-classification-of-omicron-(b.1.1.529)-sars-cov-2-variant-of-concern ).

The emergence of VOCs is concerning because these mutations may affect global epidemiology due to their high transmissibility. This poses a major threat to international public health. Concurrently, these variants impair the epidemiological blueprint of COVID-19 and have an increased virulence pattern. VOCs can alter the COVID-19 clinical manifestation [15]. Furthermore, vaccine efficacy may be uncertain because of their potential for immune evasion. The new VOCs can reduce the detection sensitivity of the RT-PCR-based diagnostic tools, especially when mutations occur at locations where probes and primers are likely to bind [53]. In addition, evidence suggests that the VOCs Alpha and Beta increased the transmissibility rate by ~ 50%, especially in younger groups and children. The Alpha variant was shown to increase hospitalisations and mortality, which may be attributed to their escape from neutralising antibodies due to their RBD mutations [54]. In view of this, immediate public health actions are required, such as controlling the transmission of SARS-CoV-2 variants, adding multiple detection methods, fast-tracking sequencing and analysis, and closely monitoring mutants and epidemiological studies to assess the effectiveness of existing or novel therapeutic drugs and vaccines against these variants.

## Characteristics of VOIs

### C.37 (Lambda)

On 15 June 2021, the lineage Lambda of SARS-CoV-2 was considered a VOI by the WHO (www.who.int/publications/m/item/weekly-epidemiological-update-on-covid-19---15-june-2021). The first patient in this lineage can be traced back to the Global Initiative on Sharing Avian Influenza Data (GISAID) in November 2020 (Lima). At present, this lineage has also been identified in the United States, Chile, Brazil, Argentina, Ecuador, Mexico, Spain, Germany, and many other countries.

The Lambda variant displays a novel deletion and multiple non-synonymous mutations in the *S* gene (Δ246-252, G75V, T76I, L452Q, F490S, D614G, R246N and T859N) (https://outbreak.info/situation-reports?pango=C.37). Among them, the mutations L452Q and F490S are located in the RBD and F490S has been associated with reduced susceptibility to antibody neutralisation [55]. At present, Lambda strain infection has been reported in at least 30 countries and regions, and the number of infections is higher in South American countries. The Lambda variant was detected in more than 70% of analysed samples in all the studied regions of Peru, except for the Amazon region, where the Gamma variant was more prevalent [56]. Official data in Peru showed that among the new confirmed cases of COVID-19 in the country, the proportion of Lambda strains was as high as 81%. This Lambda strain was the culprit responsible for the second wave of COVID-19 in the country. Recently, the Lambda strain also ‘landed’ in the Asian countries Japan and the Philippines.

The Lambda variant is under close watch because it carries numerous mutations that may increase its spread. However, there is currently a lack of definitive evidence that this variant can cause numerous serious diseases, and further research is required to determine whether this mutation really affects the behaviour of the virus.

### B.1.621 (Mu)

B.1.621 was classified as a VOI on 30 August 2021 and named ‘Mu’ by the WHO. (www.who.int/publications/m/item/weekly-epidemiological-update-on-covid-19---31-august-2021). This lineage emerged from the parental B.1 lineage that circulated in Colombia. It carries several spike mutations, some of which are common with other VOCs (D614G, E484K, N501Y, P681H), while others are new (R346K, Y144S, Y145N, D950N, and T95I) (https://outbreak.info/situation-reports?pango=B.1.621). Currently, the B.1.621 lineage is predominantly present in Colombia, the USA, Spain, the Netherlands, and Denmark [57].

As of November 26, 2021, there were two VOIs according to the WHO; they exhibit alterations in specific viral genetic markers that are predicted to enhance virulence and, therefore, remain under strict surveillance. Compared to the previous classification, B.1.617.1, B.1.526, B.1.525, P.2, P.3, and B.1.427/B.1.429 are no longer classified as VOIs. Some of them are gradually being shifted to the VUM category, as they currently do not pose a threat to the global public health.

## Characteristics of VUMs

### C.1.2

On 1 September 2021, the lineage C.1.2 of SARS-CoV-2 was classified as a VUM by the WHO (www.who.int/en/activities/tracking-SARS-CoV-2-variants/). This lineage was first discovered in the Mpumalanga and Gauteng provinces of South Africa in May 2021. It evolved from C.1, one of the dominant strains of the first wave of SARS-CoV-2 infections in South Africa and was last detected in January 2021 [58]. C.1.2 contains multiple substitutions (R190S, D215G, N484K, N501Y, H655Y and T859N) and deletions (Y144del, L242-A243del) within the spike protein (https://outbreak.info/situation-reports?pango=C.1.2), which have been observed in other VOCs and are associated with increased transmissibility and reduced neutralisation sensitivity.

### B.1.617.1

B.1.617.1 is a branch of the B.1.617 lineage, which was first detected in Maharashtra, India in late 2020/early 2021. Among the three branches of the pedigree, B.1.617.1 and B.1.617.2 accounted for 21% and 7% of the pedigree, respectively, and the B.1.617.3 sequence accounted for a limited proportion. (Available online: https://outbreak.info/(2020). This variant spread across India, causing a surge in infected individuals in the second wave of the Pandemic. In the latest weekly epidemiological report, the WHO defined B.1.617.1 as VOI, and the European Centre for Disease Control and Prevention (ECDC) classified it as VOC in May 2021. In B.1.617.1, the RBD mutated to E484Q and L452R. In addition to RBD, the important mutations of *S* glycoprotein are T95I, D614G, E154K, P681R, G142D, and Q1071H [59]. Using the double-split reporting system, Wang found that the fusion tendency of the B.1.617 mutant strain was twice that of the D614G reference strain. The increase in furan activity and cell proliferation may account for the increased transmission rate of this lineage [60].

### B.1.526

The B.1.526 SARS-CoV-2 lineage was identified in New York City (NYC), US in November 2020 [61]. The lineage was first sequenced at the end of November 2020, when it represented less than 1% of the coronavirus genome sequenced in New York City [62]. However, its prevalence has increased sharply since mid-January 2021. Through genome sequencing, it was found that B.1.526 usually substituted five other amino acid substitutions in the *S* protein: L5F, T95I, D253G, D614G, and A701V, while the mutations in RBD were S477N and E484K. Within B.1.526, E484K defined the largest sub-clade, and two distinct sub-clades were each defined by S477N; both these mutations were located in the RBD of the spike [62]. Furthermore, it is reported that the E484K mutation may alter the binding affinity and lead to a sharp increase in the number of confirmed cases of the B.1.526 mutation in NYC [61]. S477N increases the infectivity of the virus by enhancing its interaction with ACE2 [63]. Another notable feature of the B.1.526 lineage is the deletion of 112888 -11296 (NSP6 106-61 108), which is also present in the B.1.1.7, B.1.351, P.1, and B.1.525 variants [64]. The greatest threat of B.1.526 appears to be its ease of spread, with an estimated transmissibility of ~35% higher than that of non-variant viruses in direct comparison. [65]. Through pseudovirus neutralisation experiments, West (2021) proved that the B.1.526 spike mutation had an adverse effect on the recovery period and the plasma neutralization titer of the vaccinated individuals [62].

### B.1.525

The B.1.525 lineage was primarily found in Nigeria and the United Kingdom in the early days of the pandemic [66]. This lineage is also known as 20A/S.484K. It has significant mutations in the *S* protein (A67V, 69del, 70del, 144del, D614G, Q677H, and F888L), and a larger mutation (E484K) in the RBD. The two in-frame deletions at positions 69-70 and 144 were also found in the B.1.1.7 lineage and are related to enhanced infectivity and transmission [67]. The E484K mutation appears independently in multiple lineages and is associated with potential immune evasion [68]. Using the pseudovirus system, Ozer (2021) found that mutations in the spike protein gene of the B.1.525 strain promoted the entry of the virus into cells expressing the receptor ACE2 and reduced the effectiveness of the antibody [69].

## Other VUMs

On 2 June 2021, the lineage AZ.5 of SARS-CoV-2 was classified as a VUM by the WHO (www.who.int/en/activities/tracking-SARS-CoV-2-variants/). It contains multiple substitutions (D614G, P681H, T95I, D796H, and E484K) and deletion (del 144/144) within the spike protein (https://outbreak.info/situation-reports?pango=AZ.5).

Similarly, on 12 October 2021, the lineage B.1.630 of SARS-CoV-2 was also classified as a VUM. (www.who.int/en/activities/tracking-SARS-CoV-2-variants/). It contains multiple substitutions (H655Y, D614G, P9L, D950N, L452R, E484Q, T478R, C136F, and A222V) and deletion (del 144/144) within the spike protein (https://outbreak.info/situation-reports?pango=AZ.5).

And on 22 November 2021, lineage B.1.640 of SARS-CoV-2 was considered a VUM (www.who.int/en/activities/tracking-SARS-CoV-2-variants/). It contains multiple substitutions (F490R, D614G, P681H, E96Q, P9L, R346S, T859N, N394S, N501Y, Y449N, D936H, R190S, and I210T) (https://outbreak.info/situation-reports?pango=B.1.640).

## Significant mutations

### D614G

Some of the mutations were selected for changes in viral fitness, virulence, and transmissibility. A classic example is the D614G mutated variant of SARS-CoV-2 which dominants all of the VOCs and VOIs globally [70]. In early of March 2020, a non-synonymous mutation from aspartic acid (D) to glycine (G) was found in the SARS-CoV-2 spike protein 614. This variant rapidly became dominant in Europe in May 2020 [71]. All the reported VOCs (B.1.1.7, B.1.351, P.1, and B.1.617.2). The D614G substitution is often accompanied by three other mutations: the C-T mutation at position 241 in the RNA-dependent RNA polymerase gene, the synonymous C-T mutation at position 3037, and the non-synonymous C-T mutation at position 14408 in the 5’untranslated region [72]. Structural analysis of the variant strain revealed that the RBD in the G614 type S protein accounted for a higher percentage in the open conformation than in the D614 type, suggesting that the ability of the variant to bind to the ACE2 receptor was improved. Moreover, D614G changes the spike trimer hydrogen bond interactions, reorients the RBD to an ‘up’ conformation, and increases the binding and infectivity of the ACE2 receptor [73]. Plante (2021) reported that the D614G mutation might alter the viral fitness of SARS-CoV-2: Using this mutation (D614G), the SARS-CoV-2 variants are gaining viral fitness to improve replication and increase transmission [70]. Patients infected with the G614 virus did not develop more serious diseases than those infected with the D614 virus, but instead produced a greater amount of virus in the nasopharyngeal swabs. The D614G mutation mechanism uses an open conformation to bind to the ACE2 receptor, thereby promoting the ear RBD, resulting in higher virion infectivity and thermal stability. This structural change may affect antigenicity and/or viral entry [74]. Additionally, Plante et al. showed that the G614 variant maintained high infectivity at various test temperatures, indicating that the D614G mutation could improve the stability of SARS-CoV-2.

### N501Y

N501Y, a mutation of asparagine at position 501 to tyrosine (N501Y), which is a residue of the RBD-ACE2 contact region [28]. This significant mutation is related to changes in the RBD region of variants B.1.1.7, B.1.351, and P.1. The mutation was also observed in the RBD region of the VOI P.3.

Through molecular dynamics simulation (MD) and Monte Carlo (MC) sampling, Ali found that the N501Y mutation enhanced the electrostatic interaction, and a strong hydrogen bond was formed between SARS-CoV-2-T500 and ACE2-D355 near the mutation site [75]. The N501Y mutation occurred at the hACE2 binding site on sRBD. It was predicted that the N501Y mutation could enhance the binding of sRBD to hACE2 through previous SARS-CoV-2 adaptation experiments on SARS-CoV-2 in mice and high-throughput screening of all possible mutations in sRBD [76]. Recently, Starr further verified that N501Y and N501F, as well as N501W and N501V, have enhanced binding affinity between sRBD and hACE2 *in vitro* [28]. Using reverse genetics methods, Liu (2021) found that N501Y continued to increase in fitness in the upper airway replication of hamster models and primitive human airway epithelial cells [77].

The open conformation of the N501Y spike protein is associated with greater effective viral entry and infection [78]. The N501Y substitution mechanism improved the affinity of the viral spike protein to cell receptors. Khan (2021) proved that N501Y behaved similarly to the wild-type mutations. Therefore, it can be inferred that the developed vaccine may be effective against the new N501Y variant [79].

### E484K

The E484K mutation arose independently in Brazil and was identified in the Rio de Janeiro state (Southeast Brazil) in early October (2021) carried by the P.2 lineage [80]. This lineage was first detected in the B.1.351 mutation and evolved independently among infected individuals with different SARS-CoV-2 genetic backgrounds [81]. E484K is related to the changes in the RBD region of VOC B.1.1.7, B.1.351, and P.1 variants, and the mutation was also noted in the RBD region of VOI P.2, P.3, B.1.525, B.1.526, and sublineages B.1.617.1 and B.1.617.3 of the B.1.617 variant. Furthermore, E484K appeared globally in March 2020, rose significantly in October, and continued to increase in November and December 2020 [82]. Nelson (2021) conducted a series of structural analyses and found that E484K may be the most critical mutation in the current SARS-CoV-2 genome in Brazil. This created a new site for amino acid 75 hACE-2 binding. This lineage was even stronger than the binding of hACE-2 to the original 501 main site (the interface between RBD and hACE-2) [83]. Additionally, SARS-CoV-2 variants containing E484K or E484Q mutations are reported to be resistant to the neutralisation of the monoclonal antibody Bamlanivimabi *in vitro* [84]. Similarly, mutant E484K has shown to enhance the escape from neutralising antibody inhibition *in vitro*, which may be related to reduced vaccine efficacy [85]. E484K has been identified as a pivotal alternative for immune evasion because it is resistant to several monoclonal antibodies and reduces the neutralising efficacy of some polyclonal serum from convalescent and vaccinated individuals. In addition, it can increase the resistance to neutralisation by several monoclonal antibodies, while most of the rehabilitative serum and immune serum induced by mRNA vaccines show reduced inhibitory activity [86]. Jangra (2021) showed that E484K affected the binding of serum polyclonal neutralising antibodies and reduced the neutralisation efficiency of SARS-CoV-2 spike protein in low or medium IgG serum [87]. This demonstrated that the E484K mutation reduced the neutralising activity of human polyclonal serum induced by the recovery period (previous strain infection) and inoculated individuals [88].

**Figure 5.**
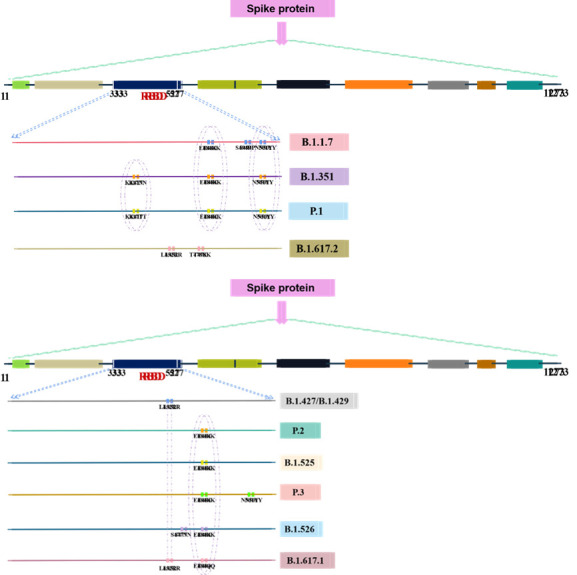
The diagram showing the position of the significant mutations in the receptor-binding domain (RBD) region in some notable mutations.

### L452R

The L452R mutation was first reported in Denmark on 17 March 2020, and it was reported in multiple states in the United States and the United Kingdom prior to 1 September 2020. This mutation was related to the changes in the RBD region of VOCs B.1.429 and B.1.427 from the United States and was also observed in the RBD region of the B.1.617 variant and its sublineages (B.1.617.1, B.1.617.2, and B.1.617.3).

Previous studies have shown that the L452R mutation may stabilise the 241 interactions between the spike protein and its human ACE2 receptor, thereby increasing infectivity [89, 90]. Deng (2021) showed that the infectivity of the L452R pseudovirus in 293T cells and human airway pulmonary organoids was higher than that of D614G, but it was slightly lower than that of the N501Y pseudovirus [91].

The replacement of L452R increased the binding affinity of SARS-CoV-2 RBD to human ACE2, protein stability and viral infectivity. Although the L452R residue is not directly located at the binding interface (Fig. 2A), structural analysis and silicon mutagenesis showed that the L452R substitution promoted electrostatic complementarity [92]. Moreover, the L452R mutation increases the stability of the *S* protein and the infectivity of the virus, thereby enhancing viral replication. The data showed that the L452R mutant escaped the cellular immunity restricted by hlaa24 and further enhanced its infectivity. A recent study showed that the B.1.427/429 variant carrying the L452R mutation was 2-6.7 times more resistant to neutralising antibodies than the non-L452R prototype virus [91].

In fact, a reduction in neutralising effects associated with L452R mutations has been reported after vaccination, although the observed neutralising antibody titre is reduced by 2.9 times [93]. A recent study revealed that the increased invasiveness of B.1.617 spike protein may be attributed to L452R itself, which can cause a 3.5-fold increase in invasiveness [59]. Another report showed that the L452R mutation reduced or eliminated the neutralising activity of 14 of 35 RBD-specific monoclonal antibodies, including three clinical-stage monoclonal antibodies [94]. Research by Motozono showed that the mutation L452R could escape the cellular immunity restricted by human leukocyte antigen (HLA) 24 and could also increase the infectivity of the virus and potentially promote virus replication [95].

### P681R

P681R is located in variant B.1.617 and belongs to all sublineages such as B.1.617.1, B.1.617.2, and B.1.617.3, and others. The P681R mutation is located near the furin cleavage site of the SARS-CoV-2 S protein (FCS; residue RRAR is located between 682-5). This substitution may affect virus replication dynamics and identify the virological characteristics of the B.1.617 variant [96]. P681R caused an increase in the alkalinity of multi-base stretching in furan cleavage, which may promote the additional contact of S1-S2 cleavage with furan. This may help to increase the rate of membrane fusion and internalisation for better transmission [97].

Bioinformatics analysis showed that the spike protein P681R mutation was highly conserved in this lineage. Although the P681R mutation reduces the infectivity of the virus, this mutation provides neutralising antibody resistance. The highly conservative P681R mutation enhances the effectiveness of viral virus fusion and further accelerates the speed of its action. The rapid kinetics of p681r-mediated viral fusion may not only be attributed to 285 immune evasion, but they may also infect exposed individuals [98].

### K417N/T

The two mutations K417T and K417N are significant mutations found in the RBD region. K417T was detected in the P.1 variant and K417N in the B.1.351 variant. The increased affinity of these two variants to ACE2 leads to an increase in the infectivity and pathogenicity of SARS-CoV-2 [99].

## Summary and perspectives

The COVID-19 pandemic is being considered the most crucial global health crisis of the century. It has caused a substantial global outbreak and is a major public health issue. The epidemic has spread worldwide, posing enormous health, economic, environmental, and social challenges to the entire human population.

The most common symptoms of coronavirus include fever, cough, tiredness, and difficulty in breathing. Initially, the individual shows mild symptoms, and in most cases, they treat it as a mild flu. The clinical spectrum for individuals with SARS-CoV-2 infection ranges from mild or non-specific signs and symptoms of acute respiratory illnesses, such as fever, cough, fatigue, dyspnoea, to severe pneumonia with respiratory failure and septic shock, which are extremely similar to other coronavirus diseases. For a respiratory disease, lung tissue damage is obvious, but other organs and tissues may also be affected due to coronavirus; in addition to the respiratory, digestive, circulatory, and genitourinary systems, the central nervous system also suffers varying degrees of damage.

The COVID-19 is a massive disaster in terms of health and economy. According to the World Trade Organization (WTO) and Organization for Economic Cooperation and Development (OECD), the COVID-19 pandemic has been the largest threat to the global economy since the financial crisis of 2008-2009. The world economic market has undergone a paradigm shift, and the share market has witnessed daily crashes. In several countries, numerous industries (except those related to infrastructure) have been closed for long periods. During the worst period of the pandemic, factories, restaurants, pubs, markets, flights, supermarkets, malls, universities, colleges and other such locations were shut down. Employment patterns have been significantly affected by the pandemic: the ‘Work from Home’ culture is being gradually endorsed by numerous institutions and individuals. The agrarian economy has also been affected, with a significant impact on food demand and security due to movement restrictions and reduced purchasing power, particularly for the most vulnerable groups. Due to the unusual outbreak of COVID-19, almost every large or small city or village in the affected countries are under partial or complete lockdown. This blockade has had a major impact on the environment. Due to the non-functioning of industries, industrial waste emissions have decreased remarkably. With substantially fewer vehicles on the road, the air quality has significantly improved. With the improvement in air quality and low environmental pollution, various birds have been spotted. In a nutshell, although COVID-19 has executed worldwide destruction, it has had a highly positive impact on the world environment.

Although pandemic prevention and control efforts have achieved remarkable results in China, SARS-CoV-2 is emerging as a global pandemic. In addition, while Wuhan was the ‘place of discovery’ of SARS-CoV-2, it was probably not the ‘place of origin’. Several recent studies have shown that there are numerous possibilities for the origin of this virus [100, 101]. As the new coronavirus continues to develop, new mutant viruses continue to emerge. Since the outbreak, in addition to strict controls based on epidemiological characteristics, researchers have been developing vaccines and targeted antiviral drugs. Enormous hope has been placed in vaccines, the development of which has progressed at an unprecedented rate throughout 2020. On 31 December 2020, the WHO listed the Comirnaty COVID-19 mRNA vaccine for emergency use, making the Pfizer-BioNTech vaccine the first to receive emergency validation since the outbreak. (www.who.int/news/item/31-12-2020-who-issues-its-first-emergency-use-validation-for-a-covid-19-vaccine-and-emphasizes-need-for-equitable-global-access (accessed on 16 June 2021). Currently, more than 100 vaccines against the SARS-CoV-2 are at various stages of development. Vaccination can be targeted at several sites on the SARS-CoV-2 surface, including unexposed nucleocapsid N, matrix protein M, envelope protein E, and the envelope spike protein [102].

Vaccine effectiveness is described as the protection provided by immunisation in a defined population. It includes both direct (vaccine-induced) and indirect (population-related) protection [103]. Preliminary research studies have revealed that an efficacy of > 70% is desired to eradicate the infection. A preventative vaccine with an efficacy of < 70% will still have a major effect and may aid in destroying the virus, given proper social distancing measures. Vaccines with an efficacy below 70% may contribute to a decrease in the length of infection [104]. Although there is a reduction in vaccine efficacy against emerging variants, vaccines can still provide considerable protection and reduce disease severity. In a test negative design study in Ontario, Canada, between December 2020 and May 2021, Nasreen (2021) estimated the effectiveness of BNT162b2 (Pfizer-BioNTech), mRNA-1273 (Moderna), and ChAdOx1 (AstraZeneca) vaccines against symptomatic SARS-CoV-2 infection and severe outcomes caused by VOCs. They thought that partial vaccination with BNT162b2 and mRNA-1273 was > 55% and > 70% effective, respectively, against symptomatic infection caused by circulating VOCs. Partial vaccination with ChAdOx1 prevented nearly half of symptomatic infections caused by Beta/Gamma and was > 60% effective against Alpha and Delta [105]. Emary (2021) showed that while laboratory neutralising antibody titres generated by vaccination with ChAdOx1 nCoV-19 vaccine were lower for the B.1.1.7 lineage, clinical vaccine efficacy against symptomatic COVID-19 was observed for the B.1.1.7 variant at 70.4%, with a lower bound of 43.6% for the 95% CI. The results showed that the ChAdOx1 nCoV-19 vaccine provided protection against symptomatic disease caused by the novel B.1.1.7 lineage. Vaccination with ChAdOx1 nCoV-19 also results in a reduction in the duration of shedding and viral load, which might reduce the transmission of the disease [106]. Madhi (2021) found that two doses of the ChAdOx1 nCoV-19 vaccine had no efficacy against the B.1.351 variant in preventing mild-to-moderate COVID-19 [107]. Hitchings (2021) confirmed that among health care workers in Manaus, the estimated effectiveness of at least one dose of the vaccine against symptomatic COVID-19 was 49.6% (95% CI 11.3-71.4) starting 14 days after the first dose of CoronaVac. A randomised controlled trial of CoronaVac in Brazil reported that the efficacy of mild, moderate and severe SARS-CoV-2 infection was 50.7% (95% CI 35.6-62.2), 83.7% (95% CI 58.0-93.7) and 100% (95% CI 564 -100) [108]. Bernal (2021) found that the absolute difference in vaccine effectiveness against symptomatic disease was approximately 12-19 percentage points for a single dose of vaccine with the Delta variant compared to the Alpha variant. The differences in vaccine effectiveness between the two doses were small. This applies to the BNT162b2 and ChAdOx1 nCoV-19 vaccines as well [109]. The Lambda variant, similar to several VOC variant spike proteins, exhibited partial resistance to neutralisation by vaccine-elicited antibodies and convalescent serum. Tada showed that the L452Q and F490S mutations of the Lambda variant spike protein caused a partial resistance to vaccine-elicited serum and regeneron monoclonal antibodies [110].

Recent research has suggested that the current vaccines will provide protection against Lambda and the other mutations discovered to date. Moreover, after an individual is completely vaccinated, the effectiveness of the vaccine is significantly improved. Complete vaccination further improves the effectiveness of serious outcomes. However, it does not preclude the possibility that existing variants will appear more resistant to the current vaccine. Perhaps as the next step in vaccine design, we should look for ways to prevent these fitness-enhancing mutations. However, for individuals, in addition to timely vaccination, the best way to prevent and hamper transmission is to protect themselves and others from infection by frequent washing of hands or using an alcohol-based rub frequently, not touching their face in public, and following social distancing norms.

How long will this epidemic last? What will be the outcome of the COVID-19? Will it gradually return like other infectious diseases and coexist with humans for a long time? The public is extremely concerned about these issues. In the last two years, the COVID-19 pandemic has been aggressive, widespread, and recurrent. It has reminded individuals of SARS and MERS, which had also taken a heavy toll on the human population. However, with effective isolation, treatment, and epidemic prevention measures, people have survived. Flu, a common infectious disease, caused approximately 25 million deaths through the ‘Spanish flu’, which exceeded the number of deaths in the World War 1. Flu can now be painlessly cured because the human body has developed antibodies during the long struggle with the flu virus, such that this highly contagious disease is unable to significantly harm the public. It is difficult to predict when a similar containment of this virus will occur, but overall, the situation is gradually growing optimistic. According to WHO statistics, the global number of COVID-19 infections and deaths is decreasing. Except for the five VOC variants, two VOI variants and several VUMs, no new variants with strong pathogenicity and infectiousness have emerged. This also indicates that there is a limit to the mutation and virulence increase of new coronaviruses during their continuous evolution, and that the virulence, pathogenicity, and infectivity of the virus may gradually decrease over time, eventually turning into a common virus that does not endanger human lives.

From another perspective, the pandemic has played a positive feedback role in public health. The resurgence of the epidemic caused by the mutant virus reflects several problems in disease prevention and control. During the pandemic, the Centers for Disease Control and Prevention, Health Commission, and hospitals continued to improve their respective responsibilities. Rapid disease screening, targeted basic treatment, and continuous updating of information have gradually normalised disease prevention. Since prehistory, and recently, from SARS to COVID-19, there has been a ceaseless struggle between humans and major infectious diseases. Such a struggle is not so much a competition as an adaptation. In this process, significant efforts have been made by scientists; from ‘unexplained pneumonia’ to the successful isolation of the diseased virus strain, various vaccines have been successfully developed and have achieved large-scale administrations in short time spans. Although the variants are evolving, scientists can discern suitable ways to control the situation. We are confident that with concerted efforts among the government, healthcare professionals, and biomedical researchers, the COVID-19 epidemic will soon be brought under control.
